# Atlantooccipital Assimilation and Basilar Invagination Treated Successfully in a Young Male With Marfanoid Features: A Stitch in Time

**DOI:** 10.7759/cureus.19365

**Published:** 2021-11-08

**Authors:** Satish Mahajan, Dhruv Talwar, Sunil Kumar, Sourya Acharya, Sandeep Iratwar, Akhilesh Annadatha

**Affiliations:** 1 Department of Medicine, Jawaharlal Nehru Medical College, Datta Meghe Institute of Medical Science (Deemed to be University), Wardha, IND; 2 Department of Neurosurgery, Jawaharlal Nehru Medical College, Datta Meghe Institute of Medical Science (Deemed to be University), Wardha, IND

**Keywords:** craniocervical abnormalities, decompression surgery, basilar invagination, atlantooccipital assimilation, marfanoid features

## Abstract

Marfan syndrome is a spectrum of disorders caused by a genetic defect involving connective tissue and is heritable by the autosomal dominant mode of inheritance. Atlantooccipital assimilation is a partial or complete fusion of the atlas and the occiput base congenitally. Although primarily asymptomatic, some patients with atlantooccipital assimilation may present with neurological issues, including myelopathy. Here, we are discussing a case of an 18-year-old male who presented with bilateral paraesthesia, tingling and neck pain which, upon investigations, turned out to be a case of atlantooccipital assimilation along with basilar invagination with spinal cord compression. The patient also had marfanoid features like tall stature, reduced upper to lower segment ratio, and increased arm span to height with positive wrist and thumb signs. As myelopathy had already developed, the patient was treated surgically rather than with medical management with a favorable outcome.

## Introduction

Marfan syndrome is a group of disorders that are caused due to genetic defects observed in the connective tissue due to mutation in the FBN1 gene on chromosome number 15, causing abnormal fibrillin protein production [1].

The skeleton of patients with Marfan syndrome typically demonstrates a wide range of deformities, which includes relatively long and thin digits known as arachnodactyly, longer limbs when compared to the length of the trunk which is known as dolichostenomelia, pectus deformities which includes two phenomena known as pectus excavatum and pectus carinatum and scoliosis of thoracolumbar vertebrae [2]. Given the broad presentation of Marfan syndrome, there is no individual finding which is diagnostic and the final diagnosis is established on the basis of clinical grounds weighing on characteristic findings. No specific lab test exists for the diagnosis of Marfan syndrome; however, the diagnosis can be facilitated with the help of molecular genetic testing. Scoliosis is the most common significant skeletal deformity seen in Marfan syndrome [3]. However, atlantooccipital assimilation with basilar invagination is a rare finding with marfanoid features.

Craniocervical abnormalities are a group of disorders that have been known for a long time. Atlas occipitalization or assimilation is an essential congenital abnormality of the junction between the cranium and vertebrae. Assimilation of the atlas indicates the fusion of the occipital bone base with the atlas, which is congenital [4]. Mostly asymptomatic, however patients with atlas assimilation may experience various symptoms such as neural compression, which is caused either by posterior arch projecting into the spinal canal or encroachment into the foramen magnum.

## Case presentation

An 18-year-old male visited the medicine outpatient department with the primary complaint of pain in the neck, weakness in the right upper and lower limb, increased sensations on bilateral upper and lower limbs and pain in both the upper and lower limbs along with tingling sensations for the last three months. There was no history of bladder and bowel involvement. There was no history of trauma, fever, or loose stools. The patient had no prior co-morbidities and there was no family history of any congenital disorder.

**Figure 1:**
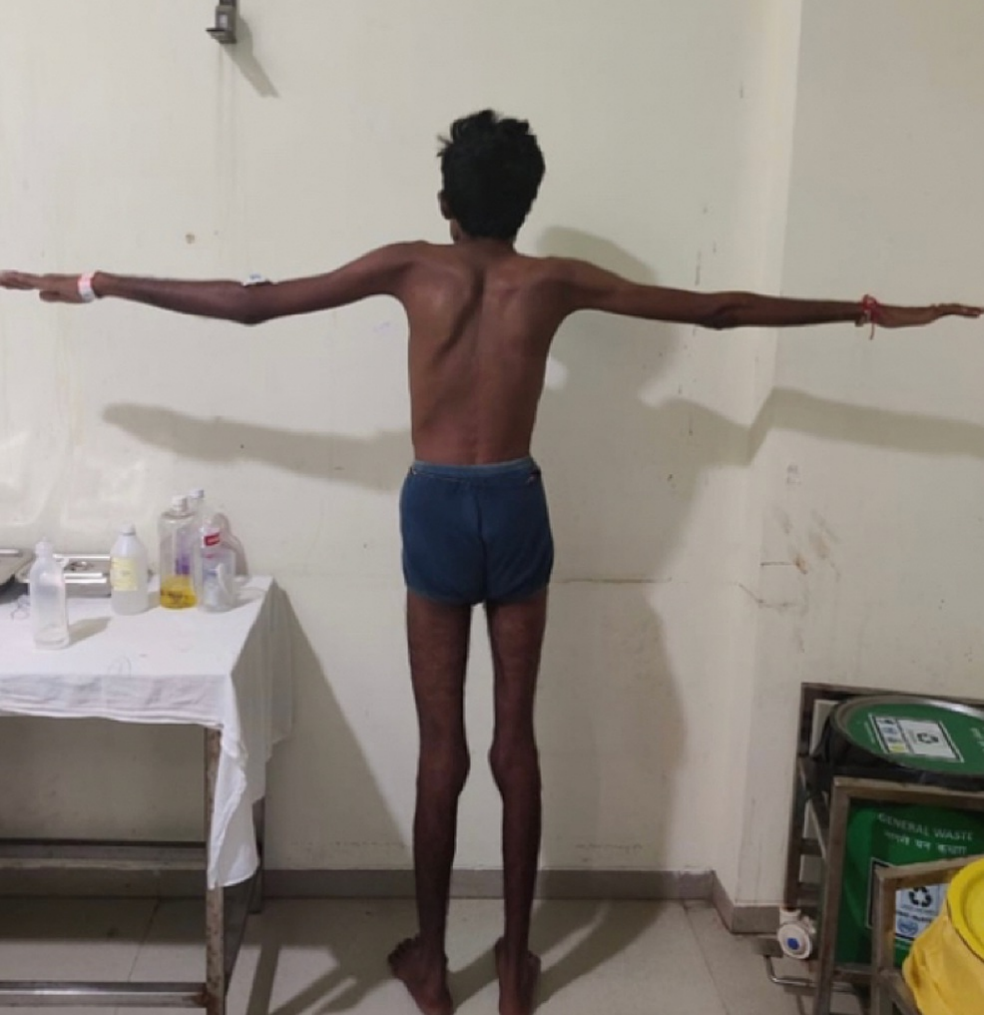
Tall stature of the patient with an increased arm span.

**Figure 2:**
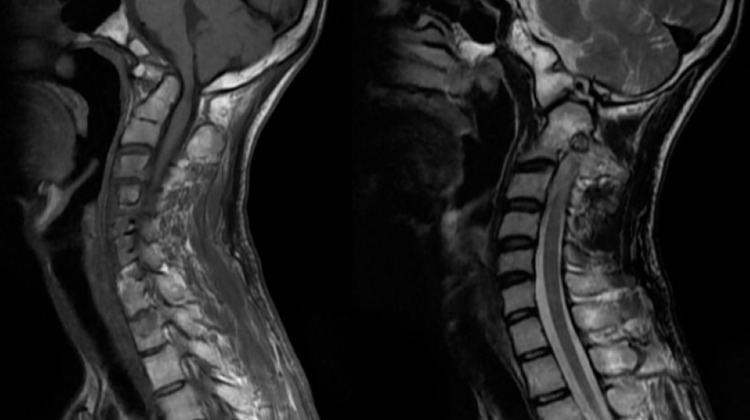
MRI cervical spine showing atlantooccipital assimilation with basilar invagination and cervical myelopathy.

**Figure 3:**
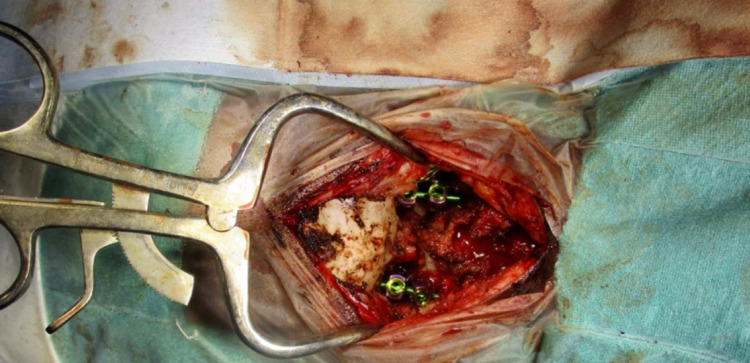
Operative procedure of decompression with cervical arthrodesis at C1-C2 level.

On examination, pulse was 88 beats per minute, regular, blood pressure was 110/70 mm Hg in right arm supine position,spo2 was 97 percent on room air, pallor was absent and there was no cyanosis. The patient had tall and thin stature with the ratio of the distance from head to pubic symphysis divided by the distance of pubic symphysis to the sole calculated to be 0.80( less than a normal ratio of 0.85) and a raised ratio of arm span length to the height calculated to be 1.09 (more than the threshold of 1.05) as shown in Figure 1. The wrist sign and thumb sign were positive. There was no low-lying hairline. The patient was conscious and oriented to, time place and person with normal higher functions. Cranial nerves were normal. On motor examination, the bulk of the muscles was bilaterally equal, the tone was normal in all four limbs, power was 4/5 in the right upper and lower limb and 5/5 in the left upper and lower limb, gait was normal and there were no involuntary movements. On sensory examination, the sensation of pain and temperature were raised bilaterally, vibration and pressure sensation were intact. Joint position and stereognosis were intact. Deep tendon reflexes were exaggerated bilaterally and were 3+ on the right upper and lower limb and 2+ on the left upper and lower limb; there was no inversion of biceps reflex and plantar was bilateral extensor. Coordination was normal. The patient had a clear chest on auscultation,s1 and s2 were heard and abdomen was soft and non-tender. MRI of the cervical spine was done, which revealed evidence of fusion of the anterior arch of the atlas with clivus along with the fusion of the posterior arch of C1 with occipital bone (Figure 2). There was hypoplasia of the occipital condyles with narrowing of the foramen magnum (8mm in anteroposterior diameter) with kinking and compression over T2 signals which were approximately 17 mm long. These features were suggestive of craniovertebral junction anomaly in the form of atlantooccipital assimilation with basilar invagination along with compression myelopathy of the adjacent cervical medullary junction and proximal cord. Routine lab investigations were carried out which were within the normal limit (Table 1). 2D echo was done, which revealed floppy mitral valve, normal chambers, good biventricular systolic function, no pericardial effusion, no signs of infective endocarditis, no mitral or aortic regurgitation and no evidence of aortic root dilatation. The patient was taken for Decompression to relieve compression along with cervical arthrodesis at C1-C2 level to provide spinal stability, as shown in Figure 3. A midline incision was made below C2 level and it was deepened, leading to exposure of subocciput, foramen magnum and C2. C2 was found to be in approximation with foramen magnum. Facet joints C1C2 were found distorted and the facet of C2 was placed superficially on the right side and the facet of C1 was deeper and had lost its normal alignment. Under C arm guidance C1C2 fixation was done and foramen magnum decompression was performed by excision of about 2.5 cm rim. The patient was given physiotherapy post-operatively. He recovered and was discharged after 23 days of surgery and is currently doing well on follow-up.

**Table 1:** Laboratory investigations of the case.

Lab parameter	Measured value
Haemoglobin	13.3gm/dl
Mean corpuscular volume	MCV-84fl
Platelet count	159000/dl
White blood cell count	6900/dl
Total protein-	7.2gm/dl
Albumin	3.6gm/dl
Globulin	3.6gm/dl
Aspartate aminotransferase	23 units/l
Alanine aminotransferase	21 units/l
Alkaline phosphatase	99 IU/l
Total bilirubin	1.1mg/dl
Creatinine	1.2mg/dl
Urea	20mg/dl
Sodium	136mmol/l
Potassium	3.8mmol/l

## Discussion

Atlantooccipitalisation or assimilation denotes an incomplete or absolute fusion occurring between the occipital bone and the atlas, which is congenital [5]. It ranges from an absolute fusion which is bony to a simple bony bridge or a band that is fibrous, causing the union of the base of the occiput to one petite portion of the atlas [6].

Occipitalisation has been reported to have an incidence of 0.08 to 3 percent of the general population and the effect is seen equally among males and females [7]. Symptoms include numbness and pain in the upper extremities, weakness, neck pain, headache and symptoms which are attributed to impingement of the posterior or anterior spinal cord or compression causing narrowing of the vertebral artery. Occipitalisation can also result in chronic atlantoaxial instability and basilar invagination, such as in our case. Patients with this condition are often reported to have short necks, low hairlines and restriction of neck movements, a finding which was missing in our case. As this abnormality varies from an absolute fusion to partial fusion, it is difficult to diagnose this condition on plain radiographs and is often missed. CT scans, tomograms and MRI are often needed to show the fusion of the occipitocervical region [8]. The patient who presents with minor symptoms or who develops symptoms only following trauma or heavy physical activity might respond to conservative management without the need for surgery. If the symptoms are not resolved by conservative or medical management, surgical decompression might be essential. If there is evidence seen radiologically showing atlantoaxial instability, decompression carried out surgically along fusion of occiput and the cervical vertebrae have been proven to provide benefits [9].

An interesting aspect of our case was the presence of marfanoid features. Although our case did not meet the diagnostic criteria of Marfan syndrome, he had certain skeletal features suggestive of marfanoid features in the absence of any other features or family history.

The involvement of the cervical spine in Marfan syndrome remains under-reported, with only a few studies contributing to the literature. A study found a large number of patients with Marfan syndrome to have increased atlantoaxial translation. It was also found that the Marfan population had increased radiological prevalence (36%) of basilar impression [10]. Also, the odontoid height was also found to be larger than normal. Cervical stenosis continues to be a rare finding in Marfan syndrome.

Our patient had already developed symptoms suggestive of myelopathy. Upon evaluation, he revealed to be a case with marfanoid features with atlantooccipital assimilation with basilar invagination. Atlantooccipital assimilation in itself is a rare finding with an incidence of about 0.5% in the general population. Its association with marfanoid features makes this finding even rarer to encounter and is being reported for the first time in the world to the best of our knowledge. Our patient was treated with the help of surgical management instead of a conservative approach which was followed by postoperative physiotherapy and ultimately resulted in complete recovery.

## Conclusions

Therefore we conclude that craniovertebral anomaly might be encountered with marfanoid features leading to the development of myelopathy. The clinicians should be aware of such rare but important presentations enabling a prompt diagnosis and management with the help of decompression surgery resulting in complete recovery. Timely diagnosis of management of this condition improves the quality of life and prevents further morbidity and mortality.
